# Comprehensive Cross-Population Analysis of High-Grade Serous Ovarian Cancer Supports No More Than Three Subtypes

**DOI:** 10.1534/g3.116.033514

**Published:** 2016-10-11

**Authors:** Gregory P. Way, James Rudd, Chen Wang, Habib Hamidi, Brooke L. Fridley, Gottfried E. Konecny, Ellen L. Goode, Casey S. Greene, Jennifer A. Doherty

**Affiliations:** * Genomics and Computational Biology Graduate Program, University of Pennsylvania, Philadelphia, Pennsylvania 19103; † Department of Systems Pharmacology and Translational Therapeutics, Perelman School of Medicine, University of Pennsylvania, Philadelphia, Pennsylvania 19103; ‡ Quantitative Biomedical Sciences, Norris Cotton Cancer Center, Geisel School of Medicine at Dartmouth, Lebanon, New Hampshire 03766; ‡ Department of Epidemiology, Geisel School of Medicine at Dartmouth, Lebanon, New Hampshire 03766; § Department of Health Sciences Research, Mayo Clinic, Rochester, Minnesota 55905; ** Department of Medicine, David Geffen School of Medicine, University of California, Los Angeles, California 90095; †† Department of Biostatistics, University of Kansas Medical Center, Kansas City, Kansas 66160; ‡‡ Department of Genetics, Geisel School of Medicine at Dartmouth, Hanover, New Hampshire 03755

**Keywords:** ovarian cancer, molecular subtypes, unsupervised clustering, reproducibility

## Abstract

Four gene expression subtypes of high-grade serous ovarian cancer (HGSC) have been previously described. In these early studies, a fraction of samples that did not fit well into the four subtype classifications were excluded. Therefore, we sought to systematically determine the concordance of transcriptomic HGSC subtypes across populations without removing any samples. We created a bioinformatics pipeline to independently cluster the five largest mRNA expression datasets using *k*-means and nonnegative matrix factorization (NMF). We summarized differential expression patterns to compare clusters across studies. While previous studies reported four subtypes, our cross-population comparison does not support four. Because these results contrast with previous reports, we attempted to reproduce analyses performed in those studies. Our results suggest that early results favoring four subtypes may have been driven by the inclusion of serous borderline tumors. In summary, our analysis suggests that either two or three, but not four, gene expression subtypes are most consistent across datasets.

Invasive ovarian cancer is a heterogeneous disease typically diagnosed at a late stage, with high mortality (Kurman and Shih 2010). The most aggressive and common histologic type is HGSC (Vang *et al.* 2009), characterized by extensive copy number variation and *TP53* mutation (Cancer Genome Atlas Research Network 2011). Given the genomic complexity of these tumors, mRNA expression can be thought of as a summary measurement of these genomic and epigenetic alterations, to the extent that the alterations influence gene expression in either the cancer or stroma.

Four gene expression subtypes with varying components of mesenchymal, proliferative, immunoreactive, and differentiated gene expression signatures have been reported in all studies of HGSC to date (Bonome *et al.* 2008; Tothill *et al.* 2008; Cancer Genome Atlas Research Network 2011; Tan *et al.* 2013; Konecny *et al.* 2014). Two of these studies also observed survival differences across subtypes (Tothill *et al.* 2008; Konecny *et al.* 2014). Tothill *et al.* (2008) first identified four HGSC subtypes (as well as two other subtypes that largely included low-grade serous and serous borderline tumors) in an Australian population using *k*-means clustering. Later, The Cancer Genome Atlas (TCGA) used NMF and also reported four subtypes that were labeled as: “mesenchymal,” “differentiated,” “proliferative,” and “immunoreactive” (Cancer Genome Atlas Research Network 2011). The TCGA group also applied NMF clustering to the Tothill data and observed similar subtypes (Cancer Genome Atlas Research Network 2011). Konecny *et al.* (2014) applied NMF to cluster an independent set of HGSC samples and reported four subtypes, which they labeled as C1–C4 (Konecny *et al.* 2014). These subtypes were similar to those in the TCGA, but a subtype classifier trained on these subtypes better differentiated survival in their own data, data from TCGA, and Bonome *et al.* (2008).

Despite the extensive research in the area, work to date has several limitations. In both the TCGA and Tothill studies, ∼8–15% of samples were excluded from analyses. A reanalysis of the TCGA data showed that over 80% of the samples could be assigned to more than one subtype (Verhaak *et al.* 2012). In more recent TCGA analyses by the Broad Institute Genome Data Analysis Center (GDAC) Firehose initiative, with the largest number of HGSC cases evaluated to date (*n* = 569), three subtypes fit the data better than four (Broad Institute TCGA Genome Data Analysis Center 2016a,b). This uncertainty in HGSC subtyping led us to determine if four homogeneous subtypes exist across study populations.

Our goal is to rigorously assess the number of HGSC subtypes. We reanalyze data from the five largest independent studies to date (and add an analysis of our own collection of samples) using a standardized bioinformatics pipeline. We apply *k*-means clustering as well as NMF to each population and do not remove “hard-to-classify” samples, as was done in previous studies (Tothill *et al.* 2008; Cancer Genome Atlas Research Network 2011). We perform independent analyses within each dataset and compare subtyping results across studies. We summarize each subtype’s expression patterns using moderated *t*-score vectors and comprehensively characterize correlations between subtypes across populations. This method contrasts with earlier reanalyses that pooled HGSC datasets together to identify subtypes (Tan *et al.* 2013). We sidestep gene expression platform or dataset biases, which could affect clustering if under or overcorrected, by comparing dataset- and subtype-specific summary statistics instead of pooling raw gene expression data.

Our cross-population comparative analysis does not support the conclusion that four HGSC subtypes exist; rather, the data more strongly support an interpretation that there are either two or three subtypes. We show that the support for four subtypes observed in TCGA’s reanalysis of the Tothill data (Cancer Genome Atlas Research Network 2011) is lost when serous borderline tumors, which have very different genomic profiles and survival compared to HGSC (Bonome *et al.* 2005; Ouellet *et al.* 2005), are excluded before clustering. Our work also highlights the impact that a single study can have on the trajectory of subtyping research and suggests the importance of periodic histopathologic review and rigorous reanalysis of existing data for cross-study commonalities.

## Materials and Methods

### Data inclusion

We applied inclusion criteria as described in detail in the supplemental materials using data from the *R* package, curatedOvarianData (Ganzfried *et al.* 2013), and our own dataset (“Mayo”). A subset of these data has been published previously (GSE53963; Konecny *et al.* 2014), but the present dataset (GSE74357) contains 343 more samples (Supplemental Material, Table S1). Briefly, these criteria selected HGSC samples from studies including at least 130 cases assayed on standard microarrays. We included only HGSC and high-grade endometrioid samples [which are molecularly similar to HGSC (Köbel *et al.* 2013)] as identified by study-specific pathological review. Data from the new Mayo HGSC samples, as well as other samples with mixed histologies and grades, for a total of 528 additional ovarian tumor samples, were deposited in NCBI’s Gene Expression Omnibus (GEO) (Edgar *et al.* 2002); these data can be accessed with the accession number GSE74357 (http://www.ncbi.nlm.nih.gov/geo/query/acc.cgi?acc=GSE74357). All study participants provided written informed consent, and this work was approved by the Mayo Clinic and Dartmouth College Institutional Review Boards.

After applying the unified inclusion criteria, our final analytic datasets included: TCGA (*n* = 499) (Cancer Genome Atlas Research Network 2011; Broad Institute TCGA Genome Data Analysis Center 2016a); Mayo (*n* = 379; GSE74357) (Konecny *et al.* 2014); Yoshihara (*n* = 256; GSE32062.GPL6480) (Yoshihara *et al.* 2012); Tothill (*n* = 242; GSE9891) (Tothill *et al.* 2008); and Bonome (*n* = 185; GSE26712) (Bonome *et al.* 2008) (Table 1). We restricted analyses to the 10,930 genes measured successfully in all five populations (Figure S1).

**Table 1:** Characteristics of the populations included in the five analytic datasets

	TCGA	Mayo	Yoshihara	Tothill	Bonome
GEO		GSE74357	GSE32062	GSE9891	GSE26712
Platform	Affymetrix HGU1133	Agilent 4x44K	Agilent 4x44K	Affymetrix HGU1133	Affymetrix HGU1133
Population	United States	United States	Japan	Australia	United States
Original sample size	578	528	260	285	195
Analytic sample size*^a^*	499	379	256	242	185
Age [Mean (SD)]	60.0 (11.6)	62.9 (11.3)	NR	60.3 (10.3)	61.5 (11.9)
Stage					
I	10 (2%)	7 (3%)	0 (0%)	11 (5%)	0 (0%)
II	17 (4%)	11 (3%)	0 (0%)	8 (4%)	0 (0%)
III	351 (80%)	275 (73%)	202 (79%)	178 (83%)	146 (80%)
IV	63 (14%)	86 (23%)	54 (21%)	17 (8%)	36 (20%)
Grade					
2	55 (12%)	3 (1%)	130 (51%)	80 (37%)	NR
3	386 (88%)*^b^*	376 (99%)	126 (49%)	134 (63%)	NR
Debulking					
Optimal	325 (74%)	287 (76%)	101 (39%)	132 (62%)	89 (49%)
Suboptimal	116 (26%)	87 (23%)	155 (61%)	82 (38%)	93 (51%)

TCGA, The Cancer Genome Atlas; NR, data not reported.

a Samples without survival data were excluded in survival analyses.

b One sample was labeled as “Grade 4” in TCGA.

### Clustering

We performed independent clustering within each dataset to avoid potential biases from different platforms or studies. As detailed in File S1, we identified the 1500 genes with the highest variance from each dataset and used the union of these genes (*n* = 3698) for clustering. We performed clustering within each dataset using each potential *k* from 2 to 4 clusters. We performed *k*-means clustering in each population using the *R* package “cluster” (version 2.0.1) (Maechler *et al.* 2014) with 20 initializations. We repeated these analyses using NMF in the *R* package “NMF” (version 0.20.5) (Brunet *et al.* 2004) with 100 different random initializations for each *k*. As done in prior studies, we calculated cophenetic correlation coefficients to select appropriate *k* for each dataset after NMF clustering with 10 consensus runs. The cophenetic correlation identifies appropriate solutions and tends to decrease with increasing *k* unless a more accurate solution is observed at a larger *k*.

### Identification of analogous clusters within and across studies

We performed significance analysis of microarray (SAM) (Tusher *et al.* 2001; Schwender *et al.* 2006) analysis on all clusters from each study using all 10,930 genes. This resulted in a cluster-specific moderated *t* statistic for each of the input genes (Schwender 2012). To summarize the expression patterns of all 10,930 genes for a specific cluster in a specific population, we combined gene-wise moderated *t* statistics into a vector of length 10,930. We repeated the SAM analysis using only the MAD subset genes and the results were similar. The TCGA subtype labels have become widely used in the field. To generate comparable labels across *k* and across studies, we mapped our TCGA subtype assignments back to the original TCGA labels to define reference clusters at *k* = 4 (that is, mesenchymal-like, proliferative-like, etc.). Clusters in other populations that were most strongly correlated with the TCGA clusters were assigned the same label.

### Clustering analysis of randomized data

Any clustering procedure is expected to induce strong correlational structure across clusters within a dataset, even if there is no true underlying structure. However, if there is no true underlying structure, clusters across datasets are not expected to be correlated. To assess this, we used the same datasets but shuffled each gene’s expression vector to disrupt the correlative structure. We performed within- and cross-study analyses of cluster identification using this set of data that were parallel to those performed using the nonrandomized data.

### Assessing the reproducibility of single-population studies

We compared our sample assignments at *k* = 2–4 to the four subtypes reported in the Tothill, TCGA, and Konecny publications (Tothill *et al.* 2008; Cancer Genome Atlas Research Network 2011; Konecny *et al.* 2014). Because the labels that were assigned in TCGA’s reanalysis of the Tothill data were not available, we performed NMF consensus clustering of Tothill’s data without removing low malignant potential (LMP) samples in order to generate labels for comparison.

### Data availability

We provide software under a permissive open source license to download the required data and reproduce our analyses (Way *et al.* 2015). Analyses were run in a Docker container, allowing the computing environment to be recreated (Boettiger 2015). Our Docker image can be pulled from: https://hub.docker.com/r/gregway/hgsc_subtypes/. This allows interested users to freely download the software, reproduce the analyses, and then build on this work. All data used in this analysis is publicly available including data we generated (accessible under GEO accession GSE74357).

## Results

### Clustering

To visually inspect the consistency and distinctness of clusters, we compared sample-by-sample correlation heatmaps. For *k* = 2–4 within each study, we observed high sample-by-sample correlations within clusters and relatively low sample-by-sample correlations across clusters (Figure S2). Clustering results using NMF were similar to *k* means results (Figure S3).

### Correlation of cluster-specific expression patterns

**Figure 1:**
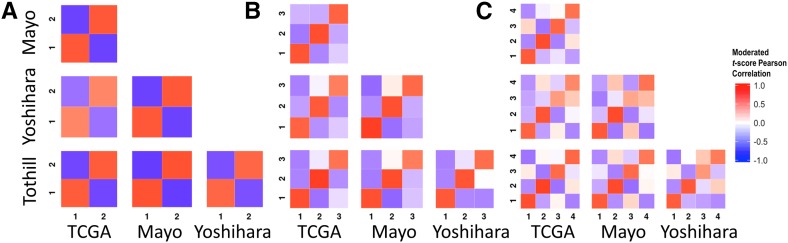
Significance analysis of microarray (SAM) moderated *t* score Pearson correlation heatmaps reveal consistency across datasets. (A) Correlations across datasets for *k* means *k* = 2. (B) Correlations across datasets for *k* means *k* = 3. (C) Correlations across datasets for *k* means *k* = 4. TCGA, The Cancer Genome Atlas.

Across datasets, we observed strong positive correlations of moderated *t* score vectors between analogous clusters in TCGA, Tothill, Mayo, and Yoshihara (Figure 1 and Table 2). However, clustering of the Bonome data did not correlate strongly with clusters identified in the other datasets (Table 2). We believe that we were unable to assign parallel subtypes in Bonome because of either RNA contamination or inappropriate grading assignments. However, more work is required in order to identify exactly why we were unable to classify. In contrast to our analyses, which independently cluster data from each study, Konecny *et al.* (2014) assigned subtypes to the Bonome data by applying a Predictive Analysis of Microarray (PAM) (Tibshirani *et al.* 2002) to their own subtypes to define reduced, subtype-specific predictive gene lists. They then assigned Bonome samples based on the highest Spearman correlation against subtype centroids (Konecny *et al.* 2014).

**Table 2:** SAM moderated *t* score vector Pearson correlations between analogous clusters across populations

	Cluster 1*^a^*	Cluster 2	Cluster 3	Cluster 4
*k* = 2*^a^*	0.62–0.81	0.62–0.81	NR	NR
*k* = 3*^a^*	0.77–0.85	0.80–0.90	0.65–0.77	NR
*k* = 4*^a^*	0.77–0.85	0.83–0.89	0.51–0.76	0.61–0.75
Bonome *k* = 2*^b^*	−0.08–0.24	−0.08–0.24	NR	NR
Bonome *k* = 3*^b^*	0.45–0.46	−0.02–0.12	0.22–0.42	NR
Bonome *k* = 4*^b^*	0.50–0.57	−0.04–0.04	0.13–0.29	0.26–0.43

TCGA, The Cancer Genome Atlas; NR, data not reported.

a Correlation ranges for TCGA, Mayo, Yoshihara, and Tothill.

b Bonome is removed from gene set analyses because of low correlating clusters.

To assess our analytical approach, we performed an analysis using randomized data. This showed that within-population correlation structure was induced by clustering, but structure between populations was not (Figure S4). The off-diagonals in this figure are close to, but not exactly, zero. Permutation induces more independent features than in real gene expression data and therefore may produce much lower correlations if structure is present in real data. Comparing Figure 1 with Figure S4, we observed much higher correlation across datasets (Figure 1), which was lost after randomization (Figure S4). For example, for *k* = 2, the TCGA and Mayo cluster correlations for analogous clusters was high (top left panel in Figure 1). Conversely, the same relationship in randomized data (second row, first column panel in Figure S4) showed correlations near zero. This indicates that the high correlations observed across datasets in Figure 1 are induced by similar underlying structure in the data.

Across studies, positive correlations between analogous clusters and negative correlations between nonanalogous clusters were stronger for clusters identified when *k* = 2 and *k* = 3 than when *k* = 4 (Figure 1), with comparable statistical precision (Table S2). These cross-population comparisons suggested that two and three subtypes fit HGSC gene expression data more consistently than the four widely accepted subtypes.

**Figure 2:**
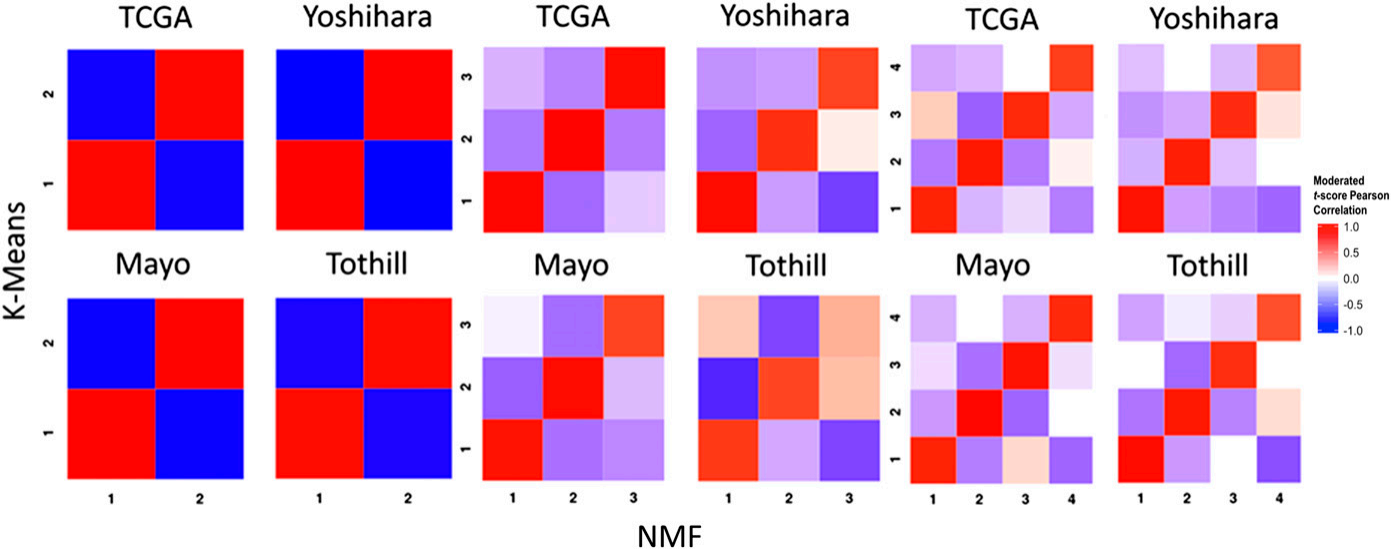
Significance analysis of microarray (SAM) moderated *t* score Pearson correlation heatmaps of clusters formed by *k* means clustering and NMF clustering reveals consistency across clustering methods. Within dataset results are shown for both methods when setting each algorithm to find 2, 3, and 4 clusters. NMF, nonnegative matrix factorization; TCGA, The Cancer Genome Atlas.

Within each population, clusters identified by NMF were similar to those identified using *k*-means clustering (Figure 2), suggesting that these results were independent of clustering algorithm. With NMF, both positive and negative correlations were stronger for *k* = 2 and *k* = 3 than for *k* = 4. Across *k* = 3 and *k* = 4, correlations were strongest for clusters 1 and 2. Sample cluster assignments for both *k*-means and NMF clusters are provided in Table S3.

### Comparison with previously-identified HGSC clusters

Our clustering results for the Tothill, TCGA, and Mayo datasets were highly concordant with the clustering described in the original publications (Tothill *et al.* 2008; Cancer Genome Atlas Research Network 2011; Konecny *et al.* 2014), as evidenced by the high degree of consistent overlap in sample assignments to the previously-defined clusters (Table 3). Our cross-study cluster 1 was mostly mapped to the “Mesenchymal” label from TCGA, “C1” from Tothill, and “C4” from Mayo. This cluster was the most stable in our analysis within all datasets, across *k* = 2, 3, and 4, and across clustering algorithms. Cross-study cluster 2, which was also observed consistently, was most similar to the “Proliferative” label from TCGA, “C5” from Tothill, and “C3” from Mayo. Cross-study cluster 3 for *k* = 3 was associated with both the “Immunoreactive” and “Differentiated” TCGA labels, “C2” and “C4” in Tothill, and “C1” and “C2” in Mayo. For analyses where *k =* 4, the third cluster was associated with “Immunoreactive,” “C2,” and “C1,” while the fourth cluster was associated with “Differentiated,” “C4,” and “C2” for TCGA, Tothill, and Mayo, respectively. For additional comparisons see the Supplementary Materials (File S1), which includes survival analyses (Table S4, Figure S9), cluster specific genes (Table S5), and pathway analyses (Table S6).

**Table 3:** Distributions of sample membership in the clusters identified in our study by the original cluster assignments in the TCGA, Tothill, and Konecny studies

	TCGA					Tothill							Konecny				
	Mes	Pro	Imm	Dif	NC	C1	C2	C3	C4	C5	C6	NC	C1	C2	C3	C4	NA
*k* = 2																	
Cluster 1	98	7	93	68	21	78	39	1	0	0	0	11	36	21	2	26	114
Cluster 2	1	127	2	60	22	0	5	5	44	35	2	22	6	39	41	0	94
*k* = 3																	
Cluster 1	98	2	20	11	6	77	22	0	0	0	0	6	16	13	2	26	82
Cluster 2	1	111	0	11	16	1	0	0	3	35	2	5	0	16	36	0	56
Cluster 3	0	21	75	106	21	0	22	6	41	0	0	22	26	31	5	0	70
*k* = 4																	
Cluster 1	97	4	12	12	5	74	0	0	0	0	0	0	7	12	3	25	62
Cluster 2	1	85	0	0	13	1	0	0	1	34	2	5	0	9	31	0	41
Cluster 3	0	5	80	3	12	3	42	0	1	1	0	14	29	6	0	1	57
Cluster 4	1	40	3	113	13	0	2	6	42	0	0	14	6	33	9	0	48

Clusters identified in our study using *k*-means clustering with *k* = 2, *k* = 3, and *k* = 4. The corresponding labels for the generally similar HGSC gene expression subtypes observed in the TCGA, Tothill, and Konecny studies are, respectively: mesenchymal/C1/C4, proliferative/C5/C3, immunoreactive/C2/C1, and differentiated/C4/C2). TCGA, The Cancer Genome Atlas; Mes, mesenchymal; Pro, proliferative; Imm, immunoreactive; Dif, differentiated; NC = samples not clustered in original publication; NA = samples not assessed at the time of the original publication.

### Meta-research into previous HGSC subtyping studies

Each of the publications that only considered high-grade samples (Cancer Genome Atlas Research Network 2011; Konecny *et al.* 2014) found clustering coefficients consistent with *k* = 2, *k* = 3, and *k* = 4. Nevertheless, each publication concludes the existence of four subtypes, while our cross-population analysis suggested that two or three clusters fit HGSC data better than four clusters.

**Figure 3:**
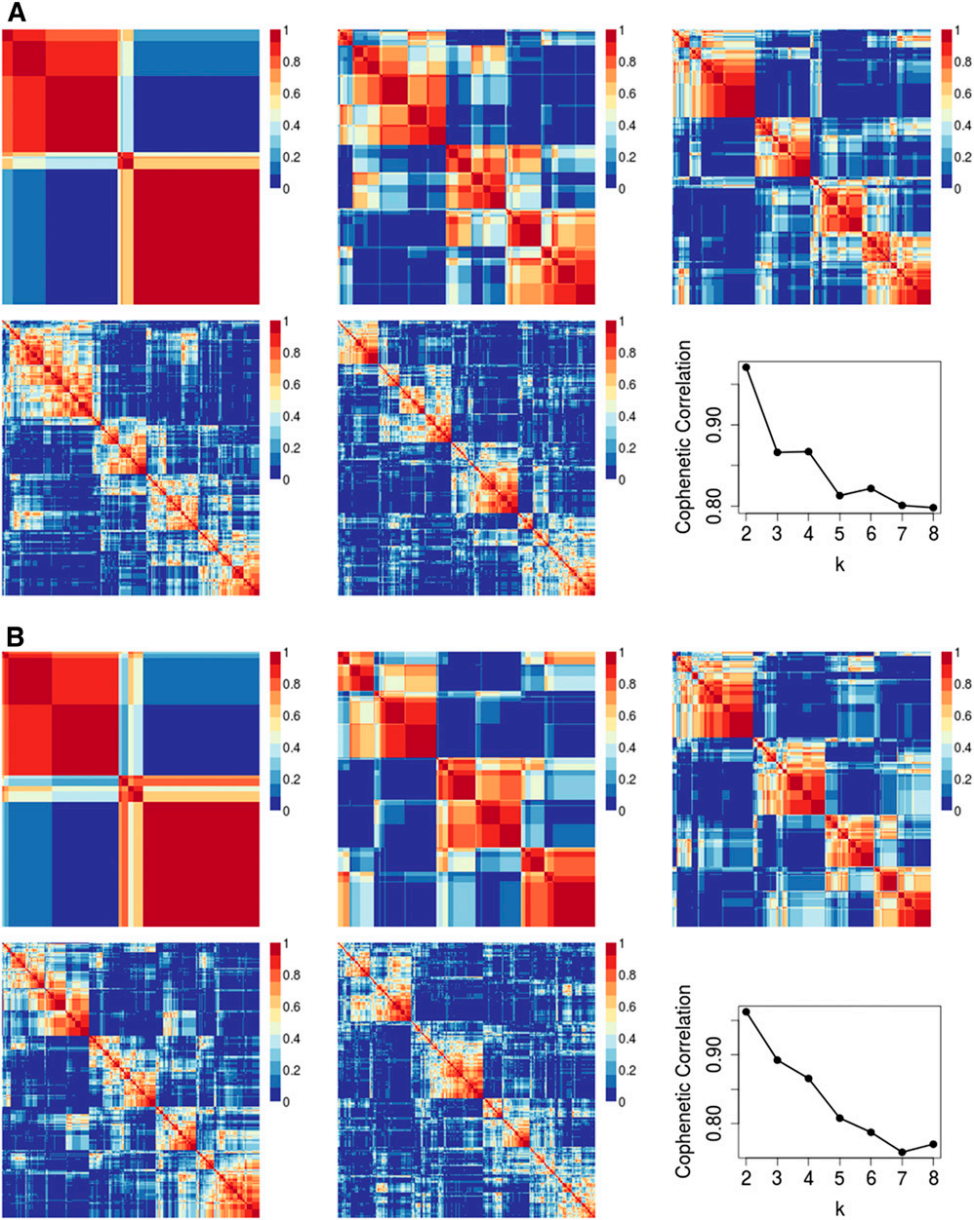
Comparing NMF consensus clustering in the Tothill dataset. Data displays consensus clustering for *k* = 2 to *k* = 6 for 10 NMF initializations alongside the cophenetic correlation results for *k* = 2 to *k* = 8. (A) Tothill dataset (*n* = 260) with borderline samples (*n* = 18) not removed prior to clustering. (B) Tothill dataset with borderline samples removed (*n* = 242).

To compare with previous results, we evaluated the number of subtypes that fit the data best within each study by calculating cophenetic correlation coefficients at *k* = 2 through *k* = 8 clusters inclusively. While cophenetic correlations typically decrease with increasing *k*, if substructure is present in the data, we would expect there to be higher values for the most appropriate number of subtypes. We observed a similar pattern in each population (Figure 3A, Figure S5, Figure S6, and Figure S7) in which the highest cophenetic correlation was reached for two clusters and, based on the heatmaps, appeared to have the highest consensus (also see Figure S8). In every dataset, four clusters were not observed to represent the data better than two or three. The only results in previous studies that contradicted this work were from TCGA’s reanalysis of the Tothill data. According to Figure S6.2 in the TCGA paper, the reanalysis included serous borderline tumors (*i.e.*, tumors with low malignant potential) (*n* = 18). The inclusion of these tumors in the TCGA HGSC reanalyses was done even though, in the original Tothill paper, the serous borderline tumors had a unique gene expression pattern and clustered entirely in a group labeled “C3.”

To assess the extent to which serous borderline tumors inclusion drove the TCGA reanalysis results, we reproduced TCGA’s reanalysis of the Tothill dataset, including the serous borderline tumors (*n* = 18); we indeed observed that the cophenetic correlation is higher for *k* = 4 than *k* = 3 (Figure 3A). However, when we appropriately removed these serous borderline tumors, we observed an increase in the *k* = 3 cophenetic correlation (Figure 3B). The results that support four subtypes were generated during clustering of HGSC and serous borderline tumors combined. Subtyping analyses of HGSC alone reveal less than four subtypes.

## Discussion

Although prior studies have reported the existence of four molecular subtypes of HGSC ovarian cancer (Cancer Genome Atlas Research Network 2011; Tothill *et al.* 2008; Konecny *et al.* 2014; Broad Institute TCGA Genome Data Analysis Center 2016a), our analysis suggests the existence of only two or three subtypes. This conclusion is based on our observation that concordance of analogous subtypes across study populations was stronger for two or three clusters as opposed to four. Previous studies used either *k*-means or NMF clustering, and because our results contradicted prior work, we performed analyses using both of these methods. Results for each population were similar for the *k* means and NMF clustering algorithms, suggesting that the clustering algorithm did not drive the observed differences.

Because cross-population comparisons suggest that two and three clusters show more consistency than four, we explored within-study heuristics (cophenetic correlation coefficients) that suggested four subtypes in previous research. The cophenetic coefficient measures how precisely a dendrogram retains sample-by-sample pairwise distances and can be used to compare clustering accuracy (Sokal and Rohlf 1962). While both the Konecny and TCGA studies reported four subtypes, in both analyses, *k* = 2 and *k* = 3 resulted in higher cophenetic coefficients than *k* = 4 [Figure 2A in Konecny *et al.* (2014) and Figure S6.1 in TCGA (Cancer Genome Atlas Research Network 2011)]. We observed the same patterns in our own reanalysis of TCGA and analysis of the expanded Mayo cohort (Figure S5 and Figure S6). Yoshihara and Tothill did not report cophenetic coefficients, but our analysis of each revealed similar patterns to TCGA and Konecny (Figure 3A, Figure S7, Figure S8, and Figure S9).

In the previous literature, the only report that suggested four subtypes represented the data better than three was TCGA’s reanalysis of the Tothill data (Figure S6.2 in their publication); the cophenetic coefficient dropped dramatically at *k* = 3 before recovering at *k* = 4 (Cancer Genome Atlas Research Network 2011). Notably, TCGA’s figure legend for this supplemental result indicates that they did not remove serous borderline tumors from the Tothill data. Our analysis of the Tothill data differed from TCGA’s in that we excluded serous borderline tumors, and instead supports the existence of two or three subtypes. To evaluate the influence of these serous borderline tumors in the Tothill data, we repeated our analyses including serous borderline tumors, and observed a drop in the cophenetic coefficient for *k* = 3 relative to *k* = 4 (Figure 3). This suggests that the four subtypes observed in TCGA’s analysis of the Tothill data may be due, in part, to the inclusion of serous borderline tumors.

There are several limitations to note in the HGSC data we analyzed. Given the intratumor heterogeneity that is likely to exist (Blagden 2015), our approach would be strengthened by having data on multiple areas of the tumors. Additionally, since histology and grade classification have changed over time (Silverberg 2000; Soslow 2008), it is unclear whether the populations we studied used comparable guidelines to determine histology and grade. We attempted to exclude all low-grade serous and low-grade endometrioid samples because they often have very different gene expression patterns and more favorable survival compared to their higher-grade counterparts (Vang *et al.* 2009). It is unclear why the Bonome clusters did not correspond to the clusters observed in other populations. Lack of consistency could result from unreported biological differences.

In summary, our study demonstrates that two clusters of HGSC, “mesenchymal-like” and “proliferative-like,” are clearly and consistently identified within and between populations. This suggests that there are two reproducible HGSC subtypes that are either etiologically distinct, or acquire phenotypically determinant alterations through their development. Our study also suggests that the previously described “immunoreactive-like” and “differentiated-like” subtypes appear to be more variable across populations, and tend to be collapsed into a single category when three subtypes are specified. These may represent, for example, steps along an immunoreactive continuum or could represent the basis of a third, but more variable, subtype. Understanding the underlying biology of the robust, well-defined “mesenchymal-like” and “proliferative-like” subtypes universally observed across populations could lead to targeted treatments that might influence survival. More work needs to be done to determine whether the heterogeneous samples that do not fall into one of these clear groups can be classified into homogeneous subtypes using other characteristics such as methylation markers or a combination of genomic measures. Our analysis reveals the importance of critically reassessing molecular subtypes across multiple large study populations using parallel analyses and consistent inclusion criteria. New systematic approaches hold promise for the implementation of such analyses (Celik *et al.* 2016; Planey and Gevaert 2016). Our results underscore the importance of ovarian cancer histopathology, contradict the four HGSC subtype hypothesis, and suggest that there may be fewer HGSC molecular subtypes with variable immunoreactivity and stromal infiltration.

## Supplementary Material

Supplemental Material
