# Supplementary material for: Comprehensive Cross-Population Analysis of High-Grade Serous Ovarian Cancer Supports No More Than Three Subtypes
Source: G3 (Bethesda). 2016 Oct 11;6(12):4097–103. doi: 10.1534/g3.116.033514 (PMC5144978; doi:10.1534/g3.116.033514)
Supplement: Supplemental Material [file supp_6_12_4097__index.html]

Comprehensive Cross-Population Analysis of High-Grade Serous Ovarian Cancer Supports No More Than Three Subtypes — Supplemental Material 

# Comprehensive Cross-Population Analysis of High-Grade Serous Ovarian Cancer Supports No More Than Three Subtypes

## Supplemental Material for Doherty *et al.*, 2016

**Files in this Data Supplement:**

- Figure S1 - Overlapping genes assayed using either the HG-U1133 Affymetrix platform (TCGA, Tothill, Bonome) or the Agilent 4x44K platform (Mayo, Yoshihara). Differences across datasets arise from inherent array differences and/or differences in quality control preprocessing. (.pdf, 168 KB)
- File S1 - Supplementary Methods (.pdf, 37 KB)
- Figure S2 - Sample by sample Pearson correlation matrices. Top panel: *k* = 2. Middle panel: *k* = 3. Bottom panel: *k* = 4. The color bars are coded as blue for cluster 1, red for cluster 2, green for cluster 3, and purple for cluster 4. In the matrices, red represents high correlation, blue low correlation, and white intermediate correlation. The scales are slightly different in each population because of different correlational structures. The clusters in the Bonome study are depicted in gray scale because in cross-population analyses to identify analogous clusters, those from Bonome did not correlate with those observed in the four other studies. (.pdf, 232 KB)
- Figure S3 - NMF consensus matrices for datasets when *k* = 2, *k* = 3, and *k* = 4. The first track represents cluster membership for k means clusters and the second track represents silhouette widths. Note that NMF clusters are not ordered in the same way as the *k* means clusters. (.pdf, 176 KB)
- Figure S4 - Significance analysis of microarray (SAM) moderated *t* score Pearson correlation heatmaps are not consistent across datasets for randomly shuffled gene expression values for *k* = 2, *k* = 3, or *k* = 4. The within dataset correlations are artificially induced because the clustering algorithm will find clusters even without true underlying structure. However, the across dataset clusters are not correlated in the randomized data indicating that the results we observe in Figure 1 are not artifacts of the clustering algorithm. (.pdf, 95 KB)
- Figure S5 - Consensus NMF clustering of the TCGA dataset (n = 499) for *k* = 2 to *k* = 6 for 10 NMF runs alongside the cophenetic correlation results for *k* = 2 to *k* = 8. (.pdf, 108 KB)
- Figure S6 - Consensus NMF clustering of the Mayo dataset (n = 379 for *k* = 2 to *k* = 6 for 10 NMF runs alongside the cophenetic correlation results for *k* = 2 to *k* = 8. (.pdf, 110 KB)
- Figure S7 - Consensus NMF clustering of the Yoshihara dataset (n = 256) for *k* = 2 to *k* = 6 for 10 NMF runs alongside the cophenetic correlation results for *k* = 2 to *k* = 8. (.pdf, 103 KB)
- Figure S8 - Silhouette width plots for *k* = 2, *k* = 3, and *k* = 4 for *k* means clustering results. Cluster 1 is shown in blue, cluster 2 in red, cluster 3 in green, and cluster 4 in purple. (.pdf, 170 KB)
- Figure S9 - Kaplan-Meier survival curves for *k* = 2, *k* = 3, and *k* = 4 shown for clustering solutions using *k* means and NMF. Cluster 1 is shown in blue, cluster 2 in red, cluster 3 in green, and cluster 4 in purple. (.pdf, 163 KB)
- Table S1 - Characteristics of Data Sets Evaluated for Inclusion. (.xlsx, 20 KB)
- Table S2 - Across dataset correlations with 95% confidence intervals. (.xlsx, 12 KB)
- Table S3 - Dataset Cluster Membership for k means and NMF. (.xlsx, 66 KB)
- Table S4 - Partial and Full Model Survival Analyses. (.xlsx, 14 KB)
- Table S5 - K means cluster specific overlapping significantly over- and underexpressed genes (TCGA, Mayo, Yoshihara, Tothill Overlap) (.xlsx, 42 KB)
- Table S6 - PANTHER Pathway analysis for differentially expressed genes in common across datasets (.xlsx, 37 KB)
