# Supplementary material for: Comprehensive Cross-Population Analysis of High-Grade Serous Ovarian Cancer Supports No More Than Three Subtypes
Source: G3 (Bethesda). 2016 Oct 11;6(12):4097–103. doi: 10.1534/g3.116.033514 (PMC5144978; doi:10.1534/g3.116.033514)
Supplement: Supplemental Material [file supp_g3.116.033514_FigureS1.pdf]

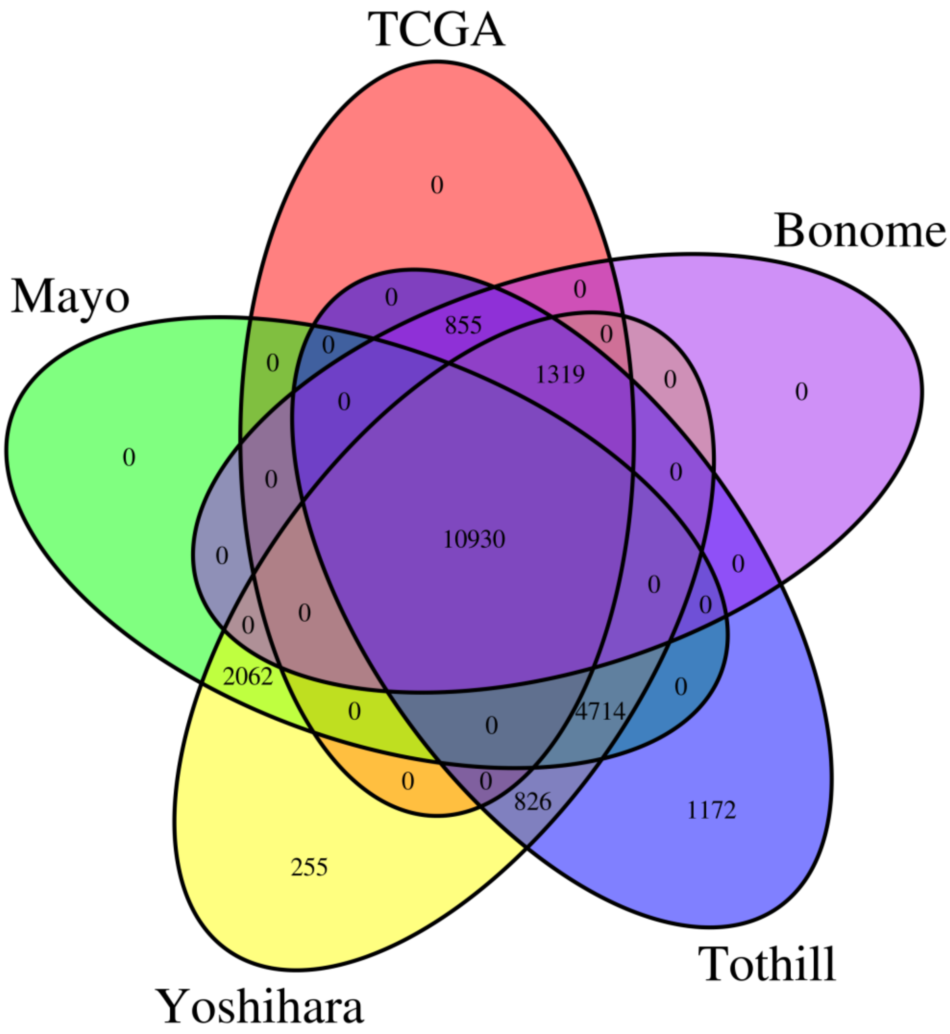

Supplementary Figure S1. Overlapping genes assayed using either the HG-U1133 Affymetrix platform (TCGA, Tothill, Bonome) or the Agilent 4x44K platform (Mayo, Yoshihara). Differences across datasets arise from inherent array differences and/or differences in quality control preprocessing.
