# Supplementary material for: Comprehensive Cross-Population Analysis of High-Grade Serous Ovarian Cancer Supports No More Than Three Subtypes
Source: G3 (Bethesda). 2016 Oct 11;6(12):4097–103. doi: 10.1534/g3.116.033514 (PMC5144978; doi:10.1534/g3.116.033514)
Supplement: Supplemental Material [file supp_g3.116.033514_FileS1.pdf]

Cross-population analysis of high-grade serous ovarian cancer does not support four subtypes

Supplementary Materials

Gregory P. Way[a,b,c], James Rudd[c,d], Chen Wang[e], Habib Hamidi[f], Brooke L. Fridley[g], Gottfried Konecny[f], Ellen L. Goode[e], Casey S. Greene[b,c,h,1], Jennifer A. Doherty[c,d,2]

a. Genomics and Computational Biology Graduate Program, University of Pennsylvania, Philadelphia, PA
b. Department of Pharmacology, Perelman School of Medicine, University of Pennsylvania, Philadelphia, PA
c. Quantitative Biomedical Sciences, Geisel School of Medicine at Dartmouth College, Lebanon, NH; Norris Cotton Cancer Center, Geisel School of Medicine at Dartmouth College, Lebanon, NH
d. Department of Epidemiology, Geisel School of Medicine at Dartmouth College, Lebanon, NH
e. Department of Health Sciences Research, Mayo Clinic, Rochester, MN
f. Department of Biostatistics, University of Kansas Medical Center, Kansas City, KS
g. Department of Medicine, David Geffen School ODF Medicine, University of California, Los Angeles, CA
h. Department of Genetics, Geisel School of Medicine at Dartmouth College, Lebanon, NH

Co-Corresponding Authors[1,2]

Casey S. Greene
10-131 SCTR 34th and Civic Center Blvd,
Philadelphia, PA 19104;
Phone: 215-573-2991;
Fax: 215-573-9135;
Email: CSGreene@upenn.edu

Jennifer A. Doherty
1 Medical Center Drive,
Lebanon, NH 03766;
Phone: 603-653-9065;
Fax: 603-653-9093;
Email: Jennifer.A.Doherty@Dartmouth.edu

1. Extended Methods

1.1 Data Inclusion

We applied the following inclusion criteria pipeline to all high-grade serous ovarian cancer datasets in the R package curatedOvarianData (v.1.3.4) (Ganzfried et al. 2013) and to an additional dataset (GSE74357; "Mayo")(Konecny et al. 2014). We first restricted to high-grade serous (grades 2 and 3) and high-grade endometrioid (grade 3) tumors, since high-grade endometrioid tumors are molecularly similar to HGSC (Kurman and Shih 2010). After these exclusions, we restricted to studies with gene expression information measured by standard microarray (Agilent or Affymetrix), and because clustering algorithms are sensitive to sample size, we included studies with at least 130 tumors (Supplementary Table S1). Our goal was to re-analyze the largest HGSC datasets and since a large portion of the analysis is comparisons across populations, including smaller, underpowered studies could inappropriately bias clustering solutions. Furthermore, to exclude duplicate samples and outliers indicating potential technical errors on these standard platforms, we used the R package "doppelgangR" (version 0.10.3) (Waldron and Riester; Waldron et al. 2014).Our final analytic datasets are described in Table 1. TCGA, Tothill, and Bonome were assayed on the HG-U133 Affymetrix platform and Yoshihara and Mayo were assayed on the Agilent 4x44K platform. Using the default mappings of probesets to gene symbols provided by the curatedOvarianData repository, we identified 10,930 genes in common (Supplementary Fig. S1). All microarray preprocessing steps were consistent for the data deposited in curatedOvarianData and are outlined in detail in Ganzfried *et al.* 2013. Processing of the Mayo data is outlined in Konecny et al. 2014.

1.2 Goodness of Fit

We identified the 1,500 genes in each population with the highest median absolute deviation (MAD), and clustered using the union of these population-specific MAD gene sets for a total of 3,698 genes. We used only use the most variable genes in this step because these genes are the most likely to be contributing to differential cluster membership and to decrease the computational cost of clustering with several random initializations. We assessed the goodness-of-fit of each clustering model using cophenetic correlation coefficients and silhouette width. The cophenetic coefficient is a measurement of how similarly the cluster dendrogram represents the Euclidean distances of all samples (Farris 1969). Additionally, we quantified certainty in subtype assignment using the silhouette width heuristic (Rousseeuw 1987). After calculating two key Euclidean distances, between a sample and its assigned cluster (distance 1) and that same sample and its closest neighbor cluster (distance 2); the silhouette width is simply the difference between distance 2 and distance 1. A sample with a negative silhouette width appears to be closer to an alternative cluster than the one to which it was assigned and may indicate low confidence in the cluster assignment. In previous studies, the *k*-means algorithm did not clearly assign all samples to specific clusters, as indicated by negative silhouette widths. While other studies removed samples with negative silhouette widths (The Cancer Genome Atlas 2011) or those that were not strongly classified, we included them to ensure that they contributed to the definition of cluster-specific differential expression

1.3 Labelling Clusters

We used significance analysis of microarray (SAM) in the R package “siggenes” (version 1.40.0; 11) on all clusters, to compare the expression patterns of the 10,930 genes in one cluster versus the expression patterns in all other clusters (within each dataset independently). This results in a moderated *t* statistic for each gene in each cluster. The statistic is a measure of the

difference in a gene's expression between samples in a given cluster compared to samples in all other clusters, weighted by the pooled variance. To compare the identified clusters across populations, we calculated Pearson correlation coefficients for every pair of moderated *t* score vectors (length of 10,930).

1.4 Survival Analyses

We evaluated whether survival differed by cluster assignment using Cox proportional hazard models (Lin and Wei 1989). Cluster assignment was modeled as either a 2, 3, or 4 level categorical variable for $k = 2$, $k = 3$, and $k = 4$ respectively. The most stable cluster within and between populations was used as the reference group (cluster 1). The full Cox model included the cluster assignment variable as well as age, stage, grade, and surgical debulking status. Since the Yoshihara data do not include age, we also created a partially adjusted model with all of the same variables except age. Age was modeled as a continuous variable. Both full and partially adjusted Cox models were created for each population using the R package "survival" (version 2.38-1; 13).

1.5 PANTHER Pathways Analyses

We identified cluster associated gene lists by taking the intersection of cluster-specific differentially expressed gene sets (at $p < 4.6\text{x}10^{-6}$) for each population (note that these differentially expressed genes were either over- or under-expressed in the specific cluster). We tested whether the gene lists were overrepresented in Gene Ontology (GO) slim pathways (Ashburner *et al.* 2000) using a Protein ANalysis THrough Evolutionary Relationships (PANTHER) pathway analysis (Mi *et al.* 2013). Using the PANTHER GO SLIM curated list of

biological process terms, we performed a binomial test for each gene list to determine over and underrepresented pathways using the 10,930 genes to define background frequencies.

1.6 Code Availability

The code used to perform all analyses and to generate figures presented in the manuscript as well as additional materials not shown in the manuscript is publically available and can be downloaded from: https://github.com/greenelab/hgsc_subtypes (Gregory Way *et al.* 2015).

2. Extended Results

2.1 Goodness of Fit

Cophenetic correlation coefficients consistently indicate a stronger clustering solution of two or three clusters as compared to four (Supplementary Figs. S5 – S7, Fig. 3B). Silhouette plots of all clusters demonstrate similar, complex patterns of clustering across datasets with cluster one consistently demonstrating the largest average silhouette width (Supplementary Fig. S8).

2.2 Survival Analysis

We visualized population specific differences in survival using Kaplan Meier survival curves (Kaplan and Meier 1958) for both k means and NMF cluster assignments (Supplementary Fig. S9). In the Mayo dataset, k = 3 cluster 3 and k = 4 cluster 4 had favorable survival (adjusted HRs and 95% confidence intervals (CI), respectively: 0.6, 0.3-0.9 and 0.4, 0.2-0.9) (Supplementary Table S4). Tothill cluster 3 in k = 4 also showed favorable survival (adjusted HR = 0.5; 95% CI = 0.3 to 0.9). In contrast to other trends, Yoshihara cluster 2 had significantly

worse survival than cluster 1 (HR = 1.9; 95% CI = 1.1 to 3.1), but these results are particularly difficult to interpret because the model could not be adjusted for age.

2.3 PANTHER Pathway Overrepresentation

Cluster associated gene lists, identified by taking the intersection of the cluster-specific differentially expressed gene sets (at $p < 4.6x10^{-6}$) for each population, are provided in Supplementary Table S5. Using each of these gene lists in a PANTHER GO slim overrepresentation analysis, we identified the biological processes terms that were significantly overrepresented (Bonferroni adjusted p-value < 0.05) (Supplementary Table S6). In general, there were several that were overrepresented in gene lists from multiple SCs. For example, the term that was most significantly overrepresented in the cluster 2 and cluster 3 gene lists for $k = 3$ was “biological adhesion”. Please refer to supplementary table S6 for a comprehensive list of overrepresented pathways.

References

Ashburner, M., C. A. Ball, J. A. Blake, D. Botstein, H. Butler *et al.*, 2000 Gene ontology: tool for the unification of biology. The Gene Ontology Consortium. Nat. Genet. 25: 25–29.

Farris, J. S., 1969 On the Cophenetic Correlation Coefficient. Syst. Zool. 18: 279.

Ganzfried, B. F., M. Riester, B. Haibe-Kains, T. Risch, S. Tyekucheva *et al.*, 2013 curatedOvarianData: clinically annotated data for the ovarian cancer transcriptome. Database 2013: bat013–bat013.

Gregory Way, James Rudd, and Casey Greene, 2015 Analytical Code for “Cross-population analysis of high-grade serous ovarian cancer reveals only two robust subtypes.”

Kaplan, E. L., and P. Meier, 1958 Nonparametric Estimation from Incomplete Observations. J. Am. Stat. Assoc. 53: 457–481.

Konecny, G. E., C. Wang, H. Hamidi, B. Winterhoff, K. R. Kalli *et al.*, 2014 Prognostic and Therapeutic Relevance of Molecular Subtypes in High-Grade Serous Ovarian Cancer. JNCI J. Natl. Cancer Inst. 106: dju249–dju249.

Kurman, R. J., and I.-M. Shih, 2010 The Origin and Pathogenesis of Epithelial Ovarian Cancer: A Proposed Unifying Theory: Am. J. Surg. Pathol. 34: 433–443.

Lin, D. Y., and L. J. Wei, 1989 The Robust Inference for the Cox Proportional Hazards Model. J. Am. Stat. Assoc. 84: 1074–1078.

Mi, H., A. Muruganujan, and P. D. Thomas, 2013 PANTHER in 2013: modeling the evolution of gene function, and other gene attributes, in the context of phylogenetic trees. Nucleic Acids Res. 41: D377–D386.

Rousseeuw, P. J., 1987 Silhouettes: A graphical aid to the interpretation and validation of cluster analysis. J. Comput. Appl. Math. 20: 53–65.

Schwender, H., A. Krause, and K. Ickstadt, 2006 Identifying interesting genes with sigenes. RNews 6: 45–50.

The Cancer Genome Atlas, 2011 Integrated genomic analyses of ovarian carcinoma. Nature 474: 609–615.

Therneau, T., 2015 A Package for Survival Analysis in S. version 2.38.:

Waldron, L., B. Haibe-Kains, A. C. Culhane, M. Riester, J. Ding *et al.*, 2014 Comparative Meta-analysis of Prognostic Gene Signatures for Late-Stage Ovarian Cancer. JNCI J. Natl. Cancer Inst. 106: dju049–dju049.

Waldron, L., and M. Riester doppelgangR: Identify likely duplicate samples from genomic or meta-data. R package version 0.8.11.:
