# Supplementary material for: Comprehensive Cross-Population Analysis of High-Grade Serous Ovarian Cancer Supports No More Than Three Subtypes
Source: G3 (Bethesda). 2016 Oct 11;6(12):4097–103. doi: 10.1534/g3.116.033514 (PMC5144978; doi:10.1534/g3.116.033514)
Supplement: Supplemental Material [file supp_g3.116.033514_FigureS2.pdf]

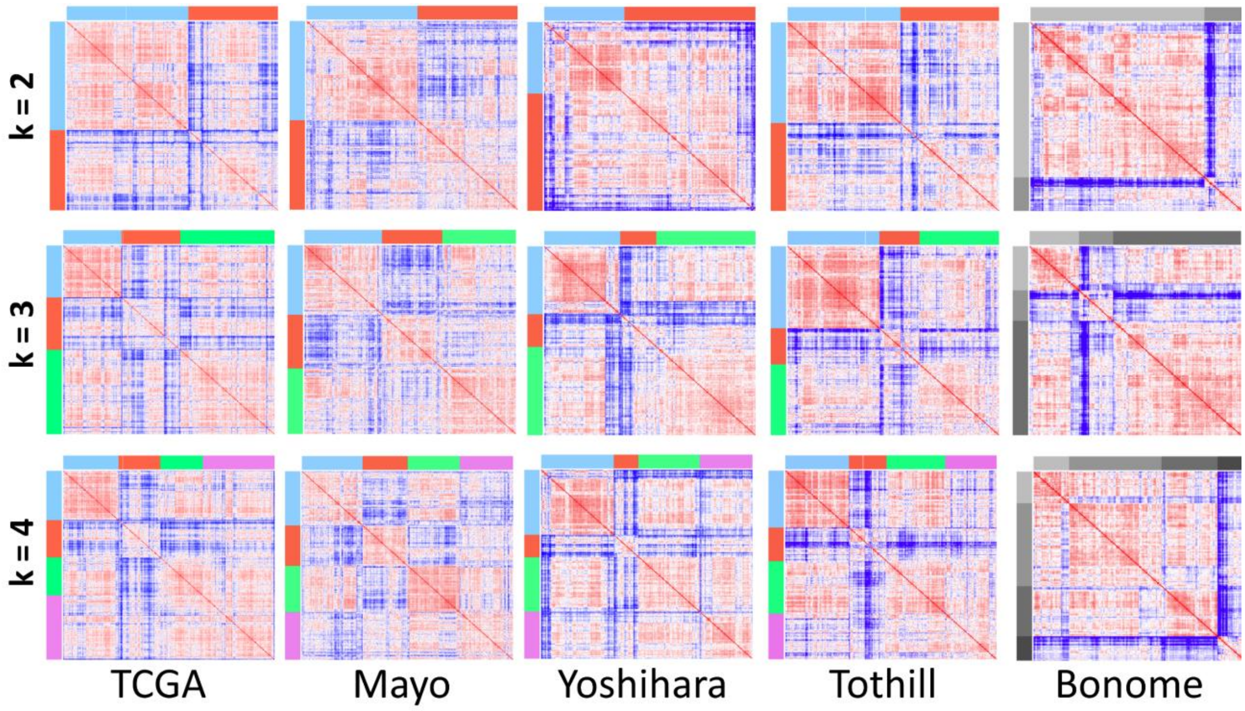

Supplementary Figure S2. Sample by sample Pearson correlation matrices. Top panel: k = 2. Middle panel: k = 3. Bottom panel: k = 4. The color bars are coded as blue for cluster 1, red for cluster 2, green for cluster 3, and purple for cluster 4. In the matrices, red represents high correlation, blue low correlation, and white intermediate correlation. The scales are slightly different in each population because of different correlational structures. The clusters in the Bonome study are depicted in gray scale because in cross-population analyses to identify analogous clusters, those from Bonome did not correlate with those observed in the four other studies.
