# Supplementary material for: Comprehensive Cross-Population Analysis of High-Grade Serous Ovarian Cancer Supports No More Than Three Subtypes
Source: G3 (Bethesda). 2016 Oct 11;6(12):4097–103. doi: 10.1534/g3.116.033514 (PMC5144978; doi:10.1534/g3.116.033514)
Supplement: Supplemental Material [file supp_g3.116.033514_FigureS3.pdf]

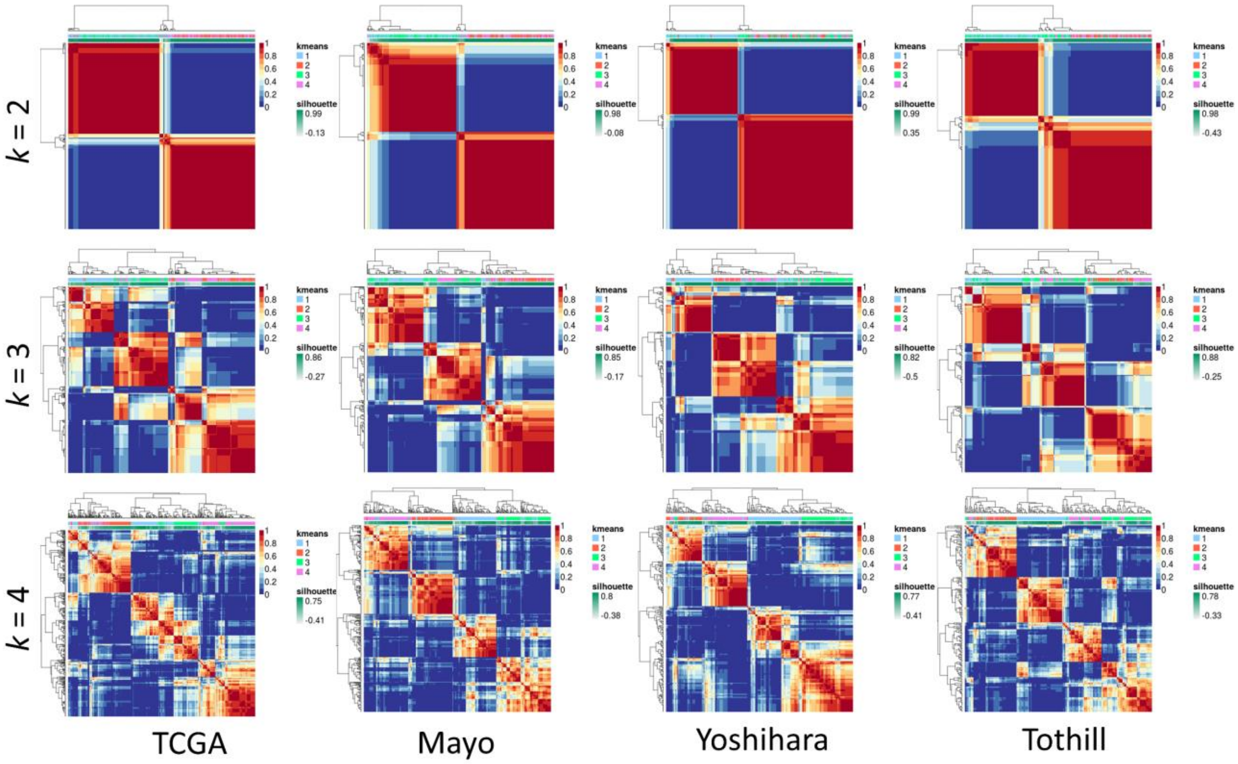

Supplementary Figure S3. NMF consensus matrices for datasets when *k* = 2, *k* = 3, and *k* = 4. The first track represents cluster membership for *k* means clusters and the second track represents silhouette widths. Note that NMF clusters are not ordered in the same way as the *k* means clusters.
