# Supplementary material for: Comprehensive Cross-Population Analysis of High-Grade Serous Ovarian Cancer Supports No More Than Three Subtypes
Source: G3 (Bethesda). 2016 Oct 11;6(12):4097–103. doi: 10.1534/g3.116.033514 (PMC5144978; doi:10.1534/g3.116.033514)
Supplement: Supplemental Material [file supp_g3.116.033514_FigureS4.pdf]

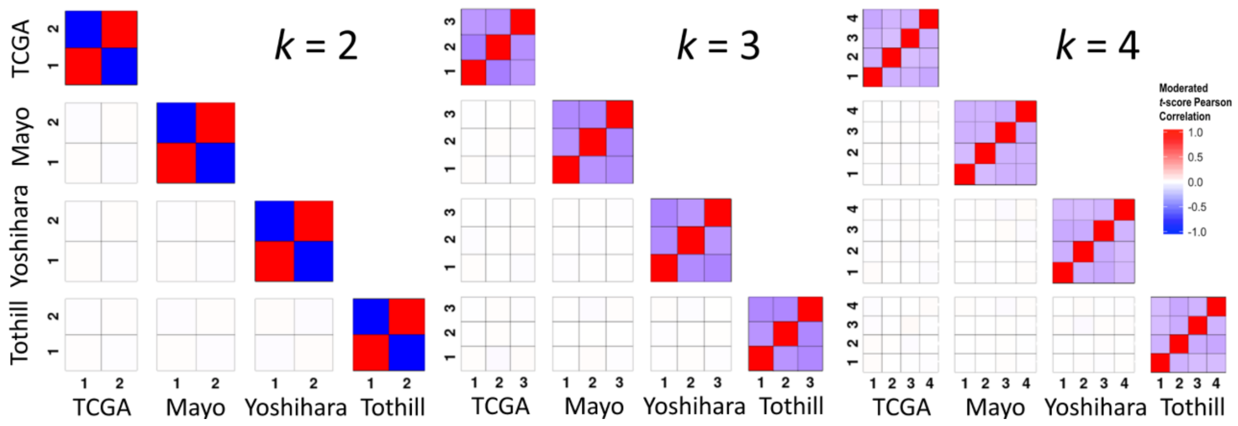

Supplementary Figure S4. Significance analysis of microarray (SAM) moderated *t* score Pearson correlation heatmaps are not consistent across datasets for randomly shuffled gene expression values for $k = 2$, $k = 3$, or $k = 4$. The within dataset correlations are artificially induced because the clustering algorithm will find clusters even without true underlying structure. However, the across dataset clusters are not correlated in the randomized data indicating that the results we observe in Figure 1 are not artifacts of the clustering algorithm.
