# Supplementary material for: Comprehensive Cross-Population Analysis of High-Grade Serous Ovarian Cancer Supports No More Than Three Subtypes
Source: G3 (Bethesda). 2016 Oct 11;6(12):4097–103. doi: 10.1534/g3.116.033514 (PMC5144978; doi:10.1534/g3.116.033514)
Supplement: Supplemental Material [file supp_g3.116.033514_FigureS6.pdf]

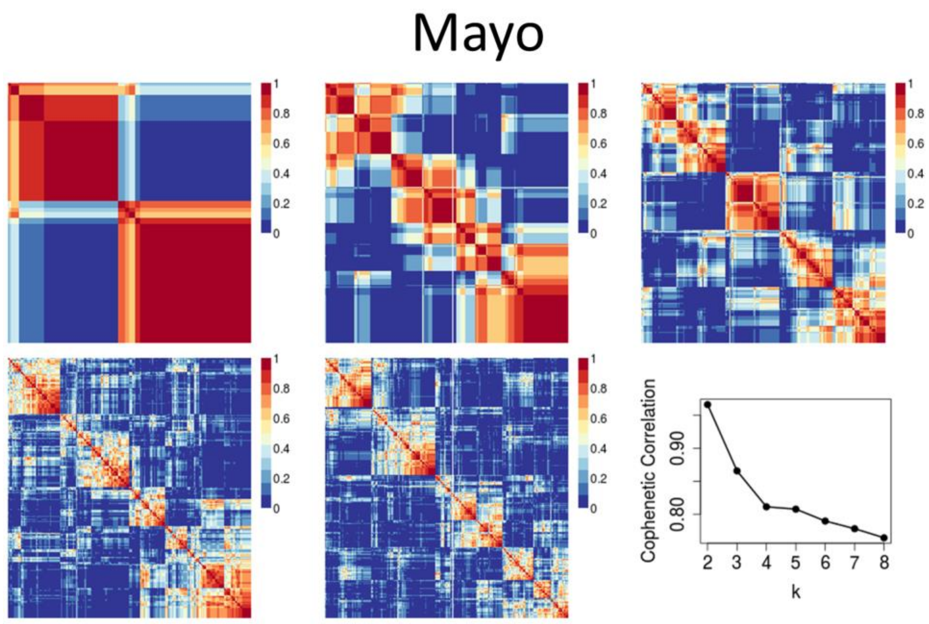

Supplementary Figure S6. Consensus NMF clustering of the Mayo dataset (n = 379 for *k* = 2 to *k* = 6 for 10 NMF runs alongside the cophenetic correlation results for *k* = 2 to *k* = 8.
