# Supplementary material for: Comprehensive Cross-Population Analysis of High-Grade Serous Ovarian Cancer Supports No More Than Three Subtypes
Source: G3 (Bethesda). 2016 Oct 11;6(12):4097–103. doi: 10.1534/g3.116.033514 (PMC5144978; doi:10.1534/g3.116.033514)
Supplement: Supplemental Material [file supp_g3.116.033514_FigureS8.pdf]

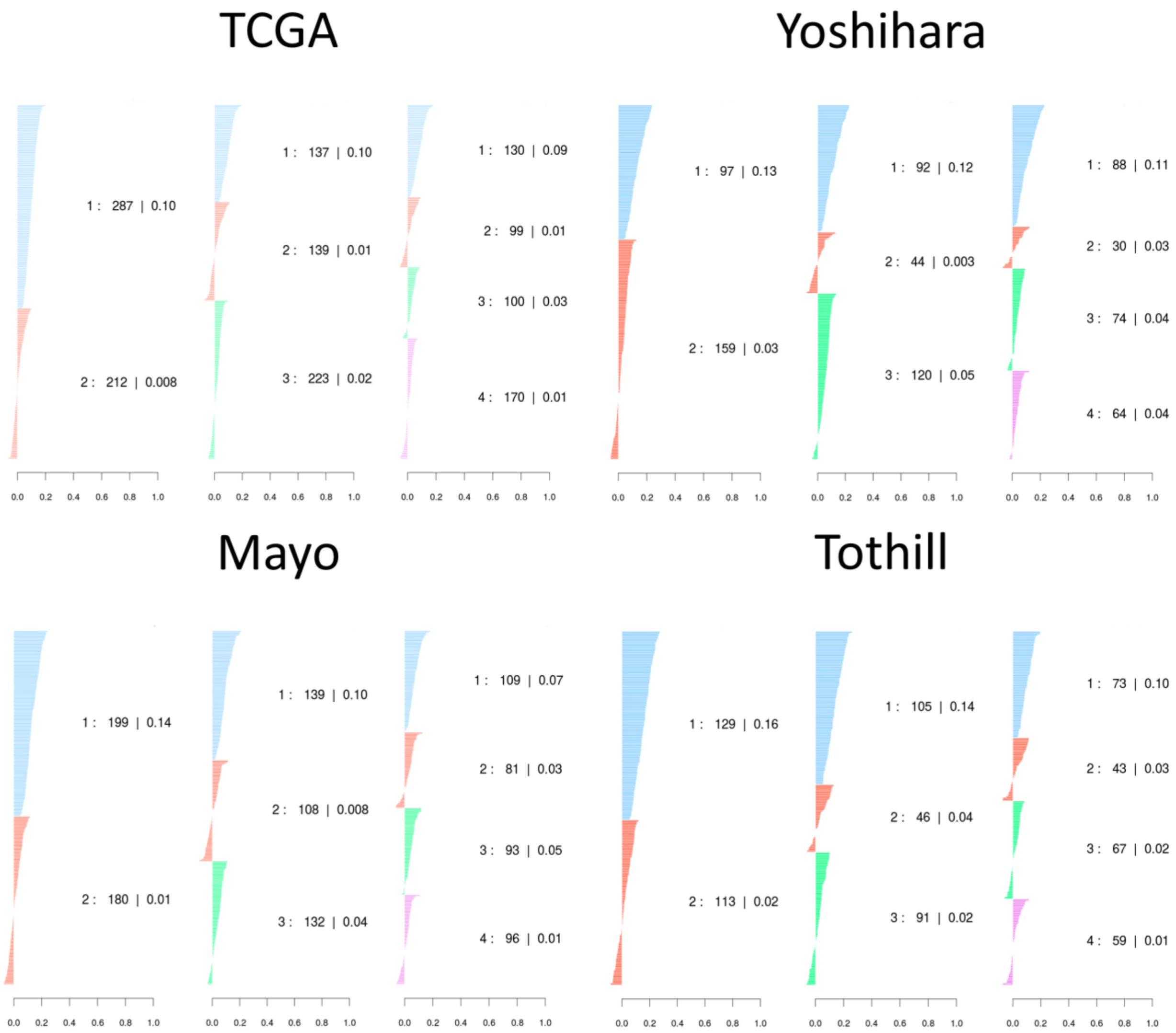

Supplementary Figure S8. Silhouette width plots for $k = 2$, $k = 3$, and $k = 4$ for k means clustering results. Cluster 1 is shown in blue, cluster 2 in red, cluster 3 in green, and cluster 4 in purple.
